# Effects of phytosterol supplementation on lipoprotein subfractions and LDL particle quality

**DOI:** 10.1038/s41598-024-61897-4

**Published:** 2024-05-15

**Authors:** Valeria Arruda Machado, Angela Rocio Niño Santisteban, Celma Muniz Martins, Nagila Raquel Teixeira Damasceno, Francisco A. Fonseca, Antonio M. Figueiredo Neto, Maria Cristina Izar

**Affiliations:** 1 Cardiology Division, Department of Medicine, Federal University of Sao Paulo, 340 - Sao Paulo, Sao Paulo, SP Brazil; 2 Institute of Physics, National Institute of Complex Fluids, University of São Paulo, São Paulo, SP Brazil; 3 Nutrition Department, Faculty of Public Health, University of São Paulo, São Paulo, SP Brazil

**Keywords:** Phytosterols, Lipoproteins, LDL-subfractions, HDL-subfractions, Dyslipidaemias, Applied physics

## Abstract

Phytosterols are natural components of plant-based foods used as supplements because of their known cholesterol-lowering effect. However, their effects on lipoprotein subfractions and the quality of the LDL particle have not been studied in greater detail. We aimed to evaluate the effects of phytosterols supplements on lipids, lipoproteins subfractions, and on the quality of LDL. A prospective, pilot-type, open label, cross-over study, randomized 23 males in primary prevention of hypercholesterolemia to receive diet or diet plus phytosterol (2.6 g in 2 doses, with meals) for 12 weeks, when treatments were switched for another 12 weeks. Lipoprotein subfractions were analyzed by electrophoresis in polyacrylamide gel (Lipoprint System®). The Sampson equation estimated the small and dense (sd) and large and buoyant (lb) LDL subfractions from the lipid profile. Quality of LDL particle was analyzed by Z-scan and UV–vis spectroscopy. Primary outcome was the comparison of diet vs. diet plus phytosterols. Secondary outcomes assessed differences between baseline, diet and diet plus phytosterol. Non-parametric statistics were performed with p < 0.05. There was a trend to reduction on HDL-7 (p = 0.05) in diet plus phytosterol arm, with no effects on the quality of LDL particles. Heatmap showed strong correlations (ρ > 0.7) between particle size by different methods with both interventions. Diet plus phytosterol reduced TC, increased HDL-c, and reduced IDL-B, whereas diet increased HDL7, and reduced IDL-B vs. baseline (p < 0.05, for all). Phytosterol supplementation demonstrated small beneficial effects on HDL-7 subfraction, compared with diet alone, without effects on the quality of LDL particles.

This trial is registered in Clinical Trials (NCT06127732) and can be accessed at https://clinicaltrials.gov.

## Introduction

Atherosclerotic cardiovascular disease (ASCVD) is characterized by chronic inflammation of the artery wall with intense oxidative process, resulting in activation of different innate cells of the immune system that are directly involved in the genesis of plaques, which are composed mainly of lipids, calcium and inflammatory cells^1^.

Dysfunctions in lipoprotein homeostasis are the biochemical basis for the development of atherosclerosis, with a direct relationship between ASCVD and increased plasma concentrations of low-density lipoprotein cholesterol (LDL-c), triglycerides and inverse relationship with high-density lipoprotein cholesterol (HDL-c) concentration^2,3^. LDL is a causal risk factor in the pathophysiology of ASCVD, and the complex physicochemical structure of LDL impacts in generation of distinct subclasses that differ in size, density, physicochemical composition, functionality, and atherogenic potential^4,5^.

Although LDL-c remains the focus for lipid-lowering therapy and lifestyle modification, high-density lipoprotein cholesterol (HDL-c) is an independent marker of ASCVD and has been included as a critical component in cardiovascular risk by both the American and European Heart Associations^6,7^. Low plasma concentrations of HDL-c have been associated with the risk of cardiovascular disease, and functionality aspects of HDL like antioxidant, anti-inflammatory, anti-thrombotic properties have also been considered^8,9^. The ability of HDL to facilitate cholesterol efflux was inversely associated with arterial intima thickness and the presence of coronary heart disease, regardless of HDL-c concentrations^10^. The efflux of cholesterol from arterial intima macrophages by HDL is crucial to prevent foam cell formation and progression of atherosclerosis^11^.

The use of food supplements with bioactive properties appears as an additional option for achieving goals or more effective reduction of LDL-c. Among these, phytosterols compete with the absorption of intestinal cholesterol, thus reducing its plasma levels. The recommended daily intake of 2 g of phytosterols reduces cholesterol absorption by between 30 and 40%, promoting a 10% decrease in LDL-c levels^12^. Phytosterols are natural components of plant-based foods used as supplements because of their known cholesterol-lowering effect^13^. They promote positive response on lipid metabolism and can be part of adjuvant treatment of individuals with different cardiovascular risk levels. However, the impact of regular use of phytosterols on qualitative characteristics of LDL and HDL is not fully clear yet.

More recently, our group validated the Z-scan (ZS) technique to investigate quality properties of LDL in relation to the balance between oxidant and antioxidant substances in this particle^14–18^. Briefly, the physical phenomenon present in these experiments is the thermal lens (TL) formed in a solution with LDL, when it is illuminated with a Gaussian laser beam. The amplitude of the TL ($\theta$), in the context of the non-linear optical study of the LDL solution, is a parameter that indicates the degree of oxidative modification of LDL, where higher values of θ indicate less modified (oxidized) LDL. However, when the thermo-optical coefficient ($dn/dT$), where $n$ and $T$ are the index of refraction and the temperature, or the linear absorption ($\alpha$) of the sample is reduced or close to zero, the modified state of the LDL particle will be bigger^19,20^. In addition to the measurement of the qualitative aspects of lipoproteins subfractions, we analyzed the oxidative profile of LDL using the innovative ZS technique applied to biological system.

The aim of this pilot-type study was to evaluate the effect of phytosterol supplementation on plasma lipids, lipoproteins subfractions and the quality of LDL particle of patients with dyslipidemia in primary prevention without drug therapy.

## Materials and methods

### Study population

**Figure 1:**
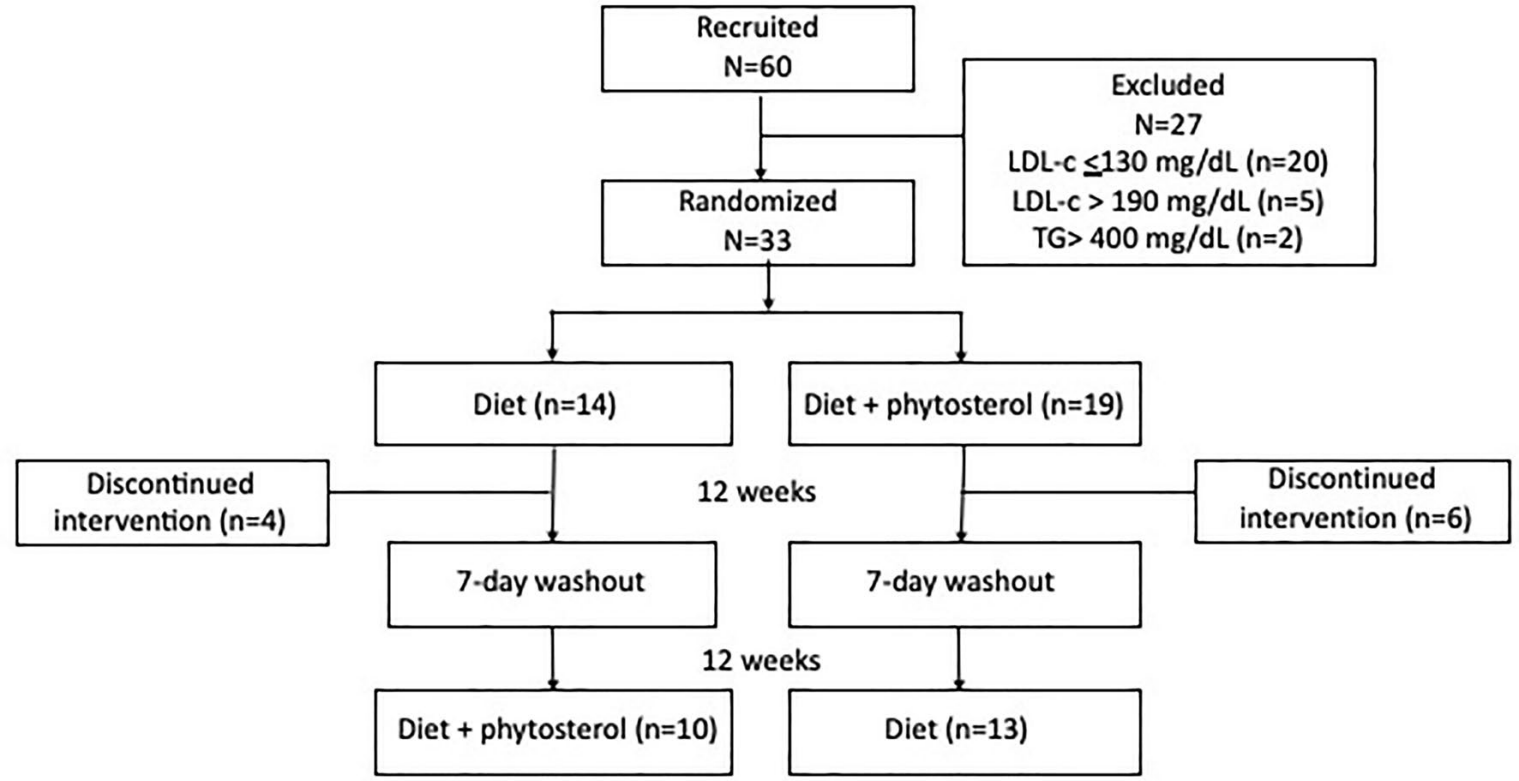
Patient disposition and study protocol.

Sixty military police individuals were selected from the Rondas Ostensivas Tobias de Aguiar (ROTA), which is a troop of the General Command of the Military Police of the State of São Paulo, Brazil, with dyslipidemia in primary prevention at low or intermediate cardiovascular risk^21,22^, without drug therapy. Twenty-seven subjects were excluded for not fulfilling inclusion criteria and 10 dropped out for not following the study protocol. The final sample consisted of 23 individuals who completed the study (Fig. 1).

The study was conducted at the Lipids, Atherosclerosis and Vascular Biology outpatient clinic, Cardiology Division, Universidade Federal de São Paulo, São Paulo, SP, Brazil. The ethical aspects followed the principles of the 1975 Declaration of Helsinki. The study protocol was approved by our local ethics committee (Comitê de Ética em Pesquisa da Universidade Federal de São Paulo, CAAE 678187.5.0000.5505, IRB 042778/2017), and all participants were included after signing the written informed consent form. This pilot trial is registered in Clinical Trials (NCT06127732) and can be accessed at https://clinicaltrials.gov.

### Inclusion/exclusion criteria

Inclusion criteria were patients of both sexes, aged > 18 years, literate, with LDL-c ≥ 130 mg/dL and < 190 mg/dL and triglycerides < 400 mg/dL.

Subjects with secondary causes of dyslipidemia, renal (creatinine > 2 mg/dL), hepatic (AST or ALT > 1.5 ULN), or metabolic (HbA1c > 8.0%) dysfunction, with BMI < 18.5 or > 40 kg/m^2^, recent surgery, disabsorptive syndrome, malignancies, under lipid-lowering therapy, unable or unwilling to participate were excluded from study protocol. Those with adherence to phytosterols < 80% were also excluded.

### Study design and data collection

This is a pilot-type prospective, randomized, open-label, crossover intervention study. We obtained a questionnaire with demographic data (sex, age), level of education, socioeconomic characteristics (self-reported income), smoking, personal and family history of dyslipidemia. Individuals who self-reported regular physical activity three times or more per week beyond daily activities, independent of type, duration and intensity were considered physically active. Information was also obtained on previous treatments with lipid-lowering agents. A general examination was performed with assessment of blood pressure and heart rate.

After the medical consultation, 12 h fasting blood samples were obtained (baseline). Subjects who met all the clinical and laboratory inclusion criteria were randomized and evaluated by the nutritionist.

### Nutrition evaluation, diet intervention and adherence

Nutritional evaluation included anthropometric data (weight, height, and waist circumference) with calculation of body mass index (BMI, kg/m^2^). Nutritional status (according to criteria recommended by the World Health Organization)^23^ and eating habits were recorded, and we provided guidance for the adoption of a healthy diet based on the Update of the Brazilian Guideline on Dyslipidemias and Prevention of Atherosclerosis^21^ and the Dietary Guide for the Brazilian population^24^. Participants were randomized into 2 groups to receive diet or diet plus phytosterols for 12 weeks, when treatments were switched for another 12 weeks.

Diet and supplements consumption, anthropometric measures, and nutritional status, were monitored by nutritionists during visits. Phytosterols (2.6 g/day) were prescribed and supplied in 650 mg gelatin capsules, composed of phytosterol esters (β-sitosterol), to be used four capsules a day along with meals, divided into 2 meals. The phytosterols (Fitocor®) were donated by Farmoquímica, Brazil.

The adherence to interventions was based in 2 strategies: I—every four weeks, follow-up visits were carried out face-to-face and adherence to phytosterol supplementation was verified by counting empty blisters, as well as dispensing the phytosterol supplement and; II – food intake estimated by three 24 h dietary recalls^25^ obtained at baseline and after four and eight weeks. The diet composition was performed by the *Food Processor* and calculation of the estimates of dietary phytosterol consumption was based in Martins et al.^26^.

### Biochemical analysis

The biochemical analyses of the study were performed at baseline, after diet and diet plus phytosterol. Total cholesterol (TC), HDL-c and triglycerides (TG) were analyzed by standard enzymatic method and automated procedures (Advia 2400, Siemens Healthcare Diagnostics, Tokyo, Japan). LDL-c was estimated by the Friedewald equation. Gamma glutamyl transpeptidase (GGT), glutamic oxaloacetic transaminase (TGO), glutamic pyruvic transaminase (TGP), creatine phosphokinase (CK), free thyroxin (FT4), and thyroid stimulating hormone (TSH) were analyzed by automated method (Advia 2400, Siemens Healthcare Diagnostics, Tokyo, Japan). Glycated hemoglobin (HbA1c) was detected by high-precision liquid chromatography (HPLC—Tosho G2, Tosho Inc., Tokyo, Japan). High-sensitivity C-reactive protein (hsCRP) was measured in plasma by nephelometry (Behringer nephelometer R100 Analyzer).

Lipoprotein subfractions (HDL and LDL) were analyzed by the Lipoprint system (Lipoprint System®; Quantimetrix, Redondo Beach, CA), which is based on the separation and quantification of lipoprotein subfractions by means of non-denaturing polyacrylamide gel. The subfractions identified in electrophoresis were integrated to determine the relative area of each lipoprotein subunit (percent of each subfraction) and, subsequently, multiplied by plasma TC concentration to calculate the amount of cholesterol in each LDL subfraction or by HDL-c to quantify the cholesterol concentration in each HDL subfraction. The method allowed the identification of seven LDL subfractions, with the LDL-1 and LDL-2 subfractions classified as large and the LDL-3 to LDL-7 subfractions as smaller and denser particles. For HDL, ten subfractions were identified, with HDL-1 to HDL-3 particles classified as large, HDL-4 to HDL-7 as intermediate and HDL-8 to HDL-10 as small.

### Low-density lipoprotein separation

LDL was isolated by serial ultracentrifugation in 2 stages, from a sample of 14 subjects at baseline, diet and diet plus phytosterol. The first step consisted of separating the VLDL production in 4.7 PC Thick-walled tubes (HIMAC), mixing 500 µL of sample, 400 µL of KBr buffer at a density of 1,019 g/mL and 1 µL of mix (5.0 µM PMSF, 10.0 µM Benzamidine, 100.0 µM BHT, 10 µM Aprotinin)^27–30^, centrifuging at 105,000 × *g*, 4 °C for 20 h in the P50AT4 fixed-angle rotor in the HIMAC CP 70MX ultracentrifuge (Hitachi®, Tokyo, Japan) and the upper phase was discarded. Later, the LDL was obtained with a second ultracentrifugation, where the contents of the tube were mixed with the same solutions and conditions as in the first separation step, but this time using KBr buffer at a density of 1,063 g/mL and recovering the upper phase^27,28^. Protein concentration was determined using a bicinchoninic acid (BCA) protein assay kit (Pierce, Rockford, IL, USA) with bovine serum albumin as standard following the manufacturer's instructions.

### Z-scan technique

The Z-scan technique was used, as previously reported, to measure non-linear optical properties of LDL samples^14–20,30,31^. The LDL solution sample (a low absorption medium) is encapsulated between two 1.5 cm × 2.0 cm glass microslides with a 200 μm thick Teflon spacer and is illuminated by a focused Gaussian laser beam with wavelength wave of 532 nm and power of 100 mW, propagating along the z direction. A sample converts light energy into heat and a thermal lens is formed on it. The thermal lens amplitude depends on the medium properties, such as thermo-optical coefficient, absorption coefficient and thermal conductivity. θ is a dimensional parameter that measures the amplitude of the TL formed in the LDL sample. In the Z-scan configuration, a mechanical chopper was used that provides a square pulse with a width of 30 *ms*, to modulate the light intensity. The transmitted light intensity is measured as a function of the z position of the sample. More details about Z-scan setup and data processing can be found in our previous works^14–20,30,31^. One can obtain the non-linear TL amplitude θ which is related to the peak-to-valley normalized transmittance as a function of the z position of the sample. As discussed before, the greater the peak-to-valley amplitude, the stronger the TL amplitude, and the less modified the LDL particle. In this study, each patient and, correspondingly, each sample had different LDL concentrations, so we normalized the values of θ to compare the results from various patients. All Z-scan experiments were performed at 37 °C.

### UV–visible spectroscopy

The linear absorbance spectra of the LDL samples were measured by a UV–visible spectrophotometer, with wavelength of light from 200 to 900 nm, using deuterium and tungsten halogen light sources and a spectrometer (USB4000, by Ocean Optics). The samples were placed in quartz cuvettes, with optical path of 1 cm. Absorbance was obtained by removing the Rayleigh scattering from the extinction spectra, measured in the spectrophotometer. In this study, we investigated the absorbance values measured at the wavelength of 484 nm, corresponding to one of the absorbance peaks of carotenoids. The maximum light absorbance of other molecules present in LDL, such as ApoB-100, cholesterol, α-tocopherol and phospholipids, show absorption peaks between 200 and 300 nm^32,33^.

### Statistical analysis

Categorical variables are presented as frequency and percentage, and continuous variables, as mean and standard deviation (SD) or median and amplitude interquartile (AIQ, Q_75%_ – Q_25%_). The normality of the distribution of numerical variables was evaluated by the Shapiro–Wilk test. For the primary outcome diet and diet plus phytosterols were compared by Mann Whitmey test. For the secondary outcome (comparisons between baseline, diet and diet plus phytosterol), we used ANOVA and post Newman-Keuls test or Friedman and post Dunn test. The Sampson equation^34^ was employed to estimate the calculated small and dense (sd) and large and buoyant (lb) LDL subfractions from the normal lipid profile, which were compared with the values obtained by Lipoprint. Heatmaps for lipid profile, HDL, and LDL subfractions, as well as the ratios of LDL subfractions (large buoyant LDL/small dense LDL) for subjects receiving diet, and diet plus phytosterol were performed. Only variables with correlation coefficient > 0.7 are shown. Spearman correlation coefficients were used, with $\rho$ variyng from -1 (blue color) to 1 (red color). The larger |*ρ*| (positive or negative), the stronger the association. All analyses were performed with the R software^34^. Values of p < 0.05 were considered statistically significant.

### Ethics approval and consent to participate

The study protocol was approved by the local ethics committee (Escola Paulista de Medicina – UNIFESP IRB 042778/2017; CAAE: 678187.5.0000.5505), which follows the latest Declaration of Helsinki, and written informed consent was provided by all patients before their inclusion.

## Results

### Demography

Patient disposition and study protocol are presented in Fig. 1. Subjects were male, age (SD) of the participants was 42 ± 6 years, and were predominantly classified as overweight, with a mean BMI of 29 ± 1 kg/m^2^. The clinical and demographic characteristics of the participants are summarized in Table 1.

**Table 1:** Clinical and demographic characteristics of study participants at baseline.

Variables	Baseline N = 23
Male (n, %)	23 (100)
Age (years)	42 ± 6
Systolic blood pressure (mmHg)	123 ± 9
Diastolic blood pressure (mmHg)	82 ± 4
Weight (kg)	95 ± 3
Waist circumference (cm)	29 ± 1
Body mass index (kg/m^2^)	97 (11)*
Income, minimum wages (%)	
4–6	7 (30)
> 6	16 (70)
Education (%)	
High School	13 (56)
Higher Education	10 (44)
Ethnicity	
White (%)	23 (100)
Smoking (%)	
Yes	1 (4)
No	22 (96)
Physical activity (%)	
Yes	17 (74)
No	6 (26)
Cardiovascular risk (%)	
Low	7 (30)
Intermediate	16 (70)

Categorical variables presented as frequencies and percentages; numerical variables presented as mean (SD) or median (AIQ).

*AIQ amplitude interquartile = Q_75%_—Q_25%_, *SD* standard deviation.

### Primary outcome: effects of phytosterol supplementation

Anthropometric variables did not differ between diet and diet plus phytosterol (Table 2), as well as biochemistry (Table 3), including the classical lipid profile.

**Table 2:** Anthropometric variables of study participants, by group.

Variable	Diet (N = 23)		Diet + phytosterol (N = 23)		p-value
	Median	AIQ	Median	AIQ	
Weight (kg)	90.5	21.85	89.4	18.65	0.99
Body mass index (kg/m^2^)	28.7	2.65	28.7	3.15	0.96
Waist circumference (cm)	94	11.85	95	10.35	0.89

Numerical variables presented as median and AIQ. Comparisons were made using Mann–Whitney test.

AIQ, amplitude interquartile = Q_75%_—Q_25%_.

**Table 3:** Biochemical profile of study participants, by group.

Variable	Diet (N = 23)		Diet + phytosterol (N = 23)		p-value
	Median	AIQ	Median	AIQ	
Glycemia, mmol/L	5.4	0.5	5.3	0.5	0.38
HbA1c, %	5.7	0.3	5.6	0.3	0.57
Total cholesterol, mmol/L	5.9	0.6	5.7	0.6	0.21
HDL-cholesterol, mmol/L	1.1	0.2	1.2	0.2	0.64
Non-HDL-cholesterol, mmol/L	4.8	0.6	4.5	0.6	0.14
LDL-cholesterol, mmol/L	4.0	0.5	3.8	0.7	0.27
Triglycerides, mmol/L	1.6	0.6	1.5	0.6	0.37
C-reactive protein, mg/L	0.19	0.19	0.24	0.22	0.40

Numerical variables presented as median and AIQ. Comparisons were made using Mann–Whitney test.

AIQ, amplitude interquartile = Q_75%_—Q_25%_

The distribution of HDL subfractions is presented in Table 4. There was a trend to lower levels of HDL7 subfraction (intermediate) in diet plus phytosterol [1.4 (1.1) vs. 1.8 (1.3), p = 0.05], when compared with diet. Table 5 shows the LDL, IDL, and VLDL subfractions, with no differences between diet and diet plus phytosterol.

**Table 4:** Distribution of high-density lipoprotein (HDL) subfractions, by group.

Variable	Diet (N = 23)		Diet + phytosterol (N = 23)		p-value
	Median	AIQ	Median	AIQ	
HDL1 (mmol/L)	0.24	0.18	0.24	0.18	0.57
HDL2 (mmol/L)	0.18	0.14	0.21	0.11	0.34
HDL3 (mmol/L)	0.12	0.05	0.13	0.09	0.19
HDL4 (mmol/L)	0.13	0.05	0.15	0.05	0.55
HDL5 (mmol/L)	0.13	0.03	0.13	0.31	0.64
HDL6 (mmol/L)	0.17	0.09	0.15	0.08	0.42
HDL7 (mmol/L)	0.05	0.03	0.04	0.03	0.05
HDL8 (mmol/L)	0.04	0.05	0.03	0.01	0.16
HDL9 (mmol/L)	0.03	0.02	0.03	0.03	0.43
HDL10 (mmol/L)	0.02	0.06	0.02	0.04	0.76
Large HDL (mmol/L)	0.55	0.16	0.53	2.72	0.68
Small HDL (mmol/L)	0.09	0.10	0.09	0.10	0.69

Numerical variables presented as median and AIQ. Comparisons were made using Mann–Whitney test.

*AIQ* amplitude interquartile = Q_75%_—Q_25%._

**Table 5:** Distribution of low-density lipoprotein (LDL) subfractions, by group.

Variable	Diet (N = 23)		Diet + phytosterol (N = 23)		p-value
	Median	AIQ	Median	AIQ	
VLDL (mmol/L)	0.80	0.32	0.80	0.21	0.78
IDLC (mmol/L)	0.39	0.14	0.39	0.14	1
IDLB (mmol/L)	0.21	0.08	0.21	0.06	0.94
IDLA (mmol/L)	0.10	0.05	0.10	0.04	0.43
LDL1 (mmol/L)	1.39	0.99	0.12	1.19	0.90
LDL2 (mmol/L)	1.65	0.05	1.44	0.48	0.15
LDL3 (mmol/L)	0.70	0.47	0.68	0.41	0.70
LDL4 (mmol/L)	0	0.32	0	0.33	0.90
LDL5 (mmol/L)	0	0	0	0	0
LDL6 (mmol/L)	0	0	0	0	0
LDL7 (mmol/L)	0	0	0	0	0
LDL size (nm)	26.5	0.7	26.4	0.9	0.83
Large LDL (mmol/L)	3.13	1.28	2.81	1.35	0.57
Small LDL (mmol/L)	0.74	0.76	0.55	0.74	0.71

Numerical variables presented as median and AIQ. Comparisons were made using Mann–Whitney test.

AIQ amplitude interquartile = Q_75%_—Q_25%._

**Figure 2:**
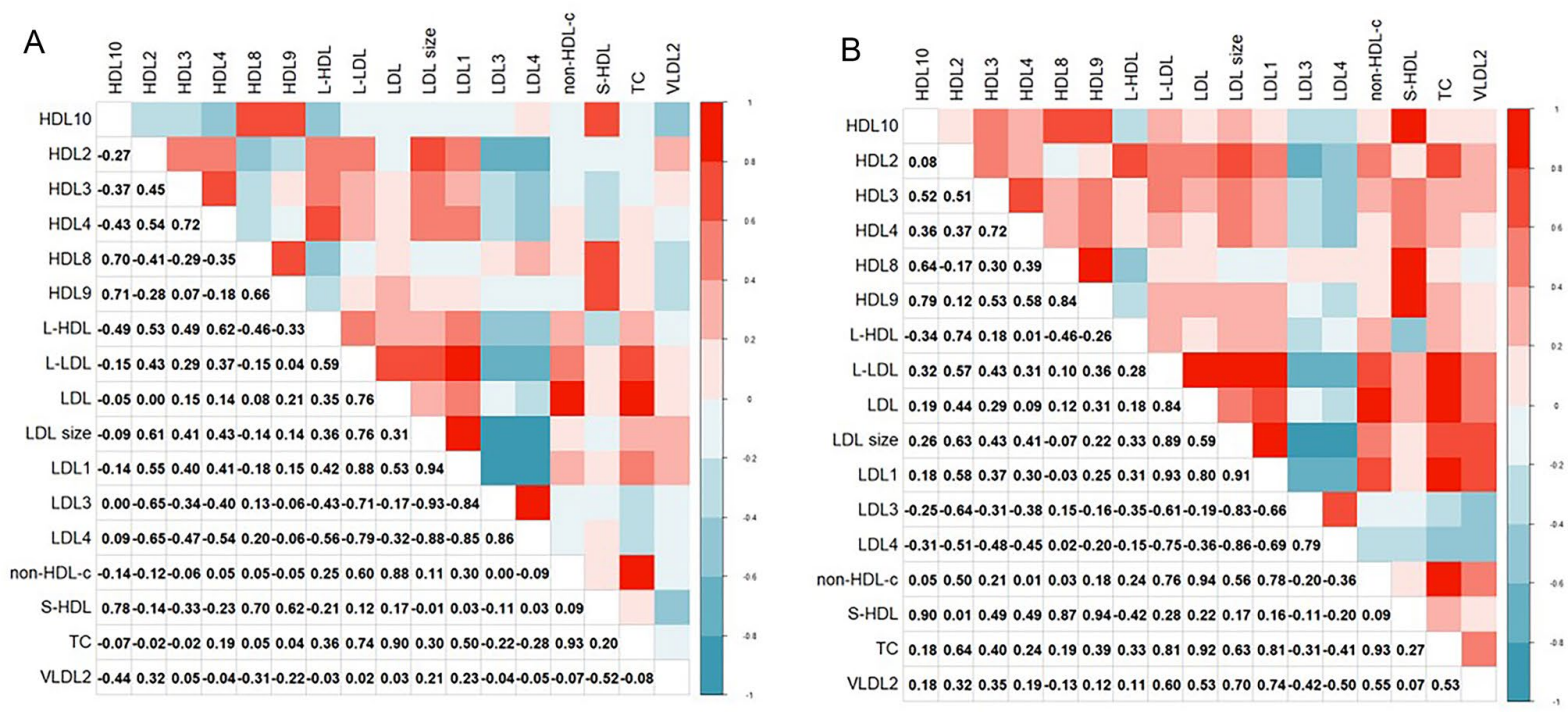
Heatmap for correlations of lipid profile, HDL, and LDL subfractions, that were significant, and with correlation coefficient* > 0.7. (**A**) Heatmap for correlations in subjects receiving diet; (**B**) Heatmap for correlations in subjects receiving diet + phytosterol. *Spearman correlation coefficient was used; ρ varies from − 1 (blue color) to 1 (red color). The larger |ρ| (positive or negative), the stronger the association. The value of the correlation coefficient is presented on the lower diagonal.

Heatmaps showing strong correlations, with |ρ| (positive or negative) > 0.07, are presented in Fig. 2 (A and B). Of note, with diet we observed strong positive correlations (ρ > 0.07) between LDL, non-HDL-c and TC; LDL1, lbLDL, and LDL size; negative correlations between LDL3, LDL4 with LDL size; LDL3 and LDL4 with LDL1.

In the diet + phytosterol intervention, strong positive correlations were observed for lbLDL, LDL1, and LDL size; HDL8, HDL9, and HDL10 with sHDL; negative correlations between LDL size, LDL3 and LDL4.

### Secondary outcome: comparisons between baseline, diet and diet plus phytosterols

Total cholesterol was lower and HDL-c higher in diet plus phytosterol vs. baseline (Supplemental Table 1); total energy intake was lower in diet and diet plus phytosterol vs. baseline (p < 0.05, for all). Diet phytosterol intake, obtained from 24-h dietary recalls was low (25 ± 18 vs. 30 ± 14 vs. 33 ± 21 mg/day), and did not differ between groups (p = 0.23) (data not shown).

Lower HDL7 was seen at baseline vs. diet (p = 0.007), IDL-B was higher at baseline, compared with diet or diet + phytosterol (p < 0.05), and there was a trend to lower sdLDL at both diet and diet + phytosterol, compared with baseline (data not shown).

### Z-scan and UV–vis spectroscopy

**Figure 3:**
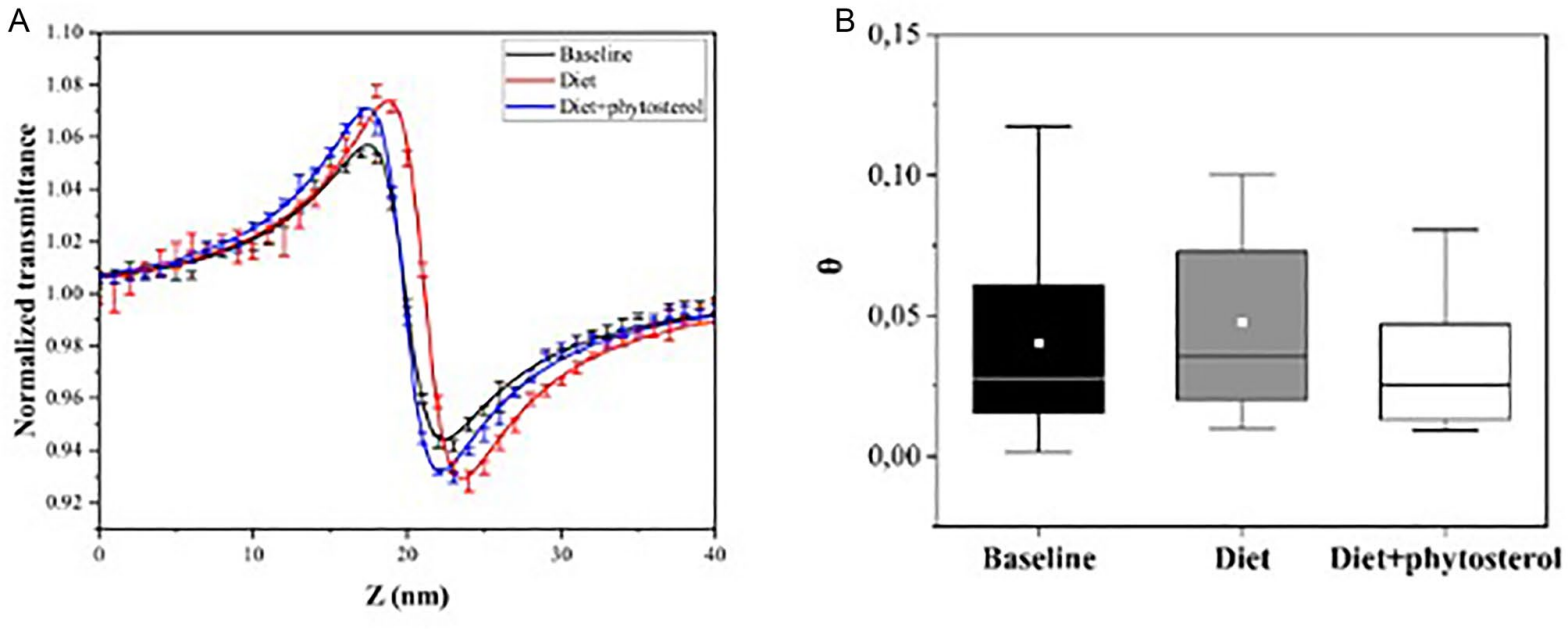
(**A**) Z Scan typical result: Normalized transmittance as a function of the sample z position of a baseline (black), diet (red) and diet + phytosterol (blue) groups; (**B**) Boxplot for phase shift (θ) at baseline, diet, and diet + phytosterol with the mean data of each group. Experimental data (symbols) and Model fits (lines). The mean of each group is in white frames inside the boxes, baseline (0.04 ± 0.03), diet (0.05 ± 0.03) and diet + phytosterol (0.03 ± 0.02), (p = 0.43).

**Figure 4:**
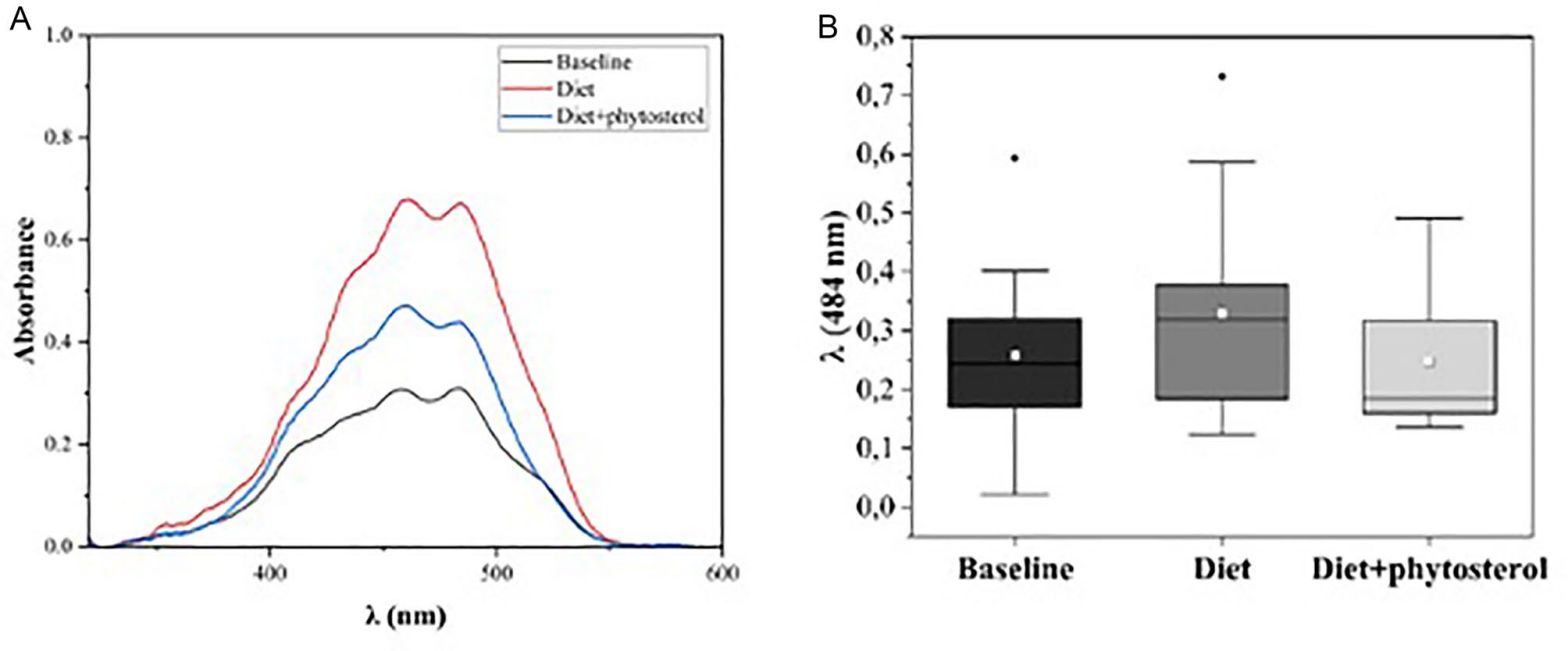
(**A**) Typical absorbance spectrum of an LDL-c sample of a baseline (black), diet (red) and diet + phytosterol (blue) groups; (**B**) Mean values of absorbance at λ = 484 nm, in the contrasting times, from the two groups. The mean of each group is in white frames inside the boxes, baseline (0.26 ± 0.13), diet (0.33 ± 0.18) and diet + phytosterol (0.24 ± 0.12).

The Z-scan and UV–vis spectroscopy are shown in Figs. 3–4. Typical Z-scan results for LDL in solution for the three groups are shown in Fig. 3A. In the 2 groups (Fig. 3B), the difference of the θ value means indicates that there was no statistically significant difference between baseline and diet or diet + phytosterol (p = 0.43). The θ values means and the linear light absorption at 532 nm showed no significant differences (p = 0.06) between baseline (0.05 ± 0.02), diet (0.06 ± 0.03) and diet + phytosterol (0.04 ± 0.02).

From the absorbance spectrum (Fig. 4A) we extracted the values at 484 nm, and a comparison between the mean values is shown in Fig. 4B. The values obtained for the absorbance of interest were not significantly different between diet or diet + phytosterol (p = 0.409). However, a strong positive correlation between θ and ${\lambda }_{484}$ was observed (r = 0.92 p < 0.0001), when all groups were considered (Supplemental Fig. 1). In Supplemental Fig. 2 we show the UV-visual spectroscopy wavelength absorbance spectrum of β-sitosterol in ethyl acetate as solvent. The spectrum at 180–400 nm corresponds to the β-sitosterol homologous molecule of cholesterol, at dilutions ranging from 0 to 2 g / mL.

## Discussion

This is a pilot study addressing effects of phytosterol intervention on lipoprotein subfractions beyond the cholesterol-lowering effect. It was carried out with military police individuals selected from the ROTA, a special battalion of armed policeman that face danger every day, without time for meals, or leisure. The primary outcome comparing interventions (diet vs. diet plus phytosterol), showed no differences for anthropometric variables, classical lipid profile, or LDL subfractions. There was a trend for lower HDL7, of intermediate size, in diet + phytosterol compared with diet (p = 0.05). These dietary interventions did not affect the quality of LDL particles. We used different ways to assess lipoprotein particle size, and the heatmaps presented some strong correlations among them.

Secondary outcomes showed that phytosterol supplementation promoted small, but favorable changes in lipids and lipoproteins subfractions, including decrease in TC, and increase in HDL-c, compared with baseline. Furthermore, both interventions (diet and diet + phytosterols), reduced the atherogenic IDLB.

Guidelines such as those of the Brazilian Society of Cardiology^21^, and the European Society of Cardiology^7^, recommend a combination of dietary and lifestyle changes as a strategy to reduce the risk of ASCVD. In addition, they recommend the inclusion of phytosterols (2 g/day) to the diet in primary prevention for patients with elevated LDL-c. Phytosterols are known dietary adjuncts that lower LDL-c without producing any apparent side effects^35^, and are indicated for individuals with high plasma LDL-c at low or intermediate cardiovascular risk, not qualified for pharmacological treatment^35–37^.

There is an inverse relationship between the usual consumption of phytosterols in the diet and serum cholesterol or LDL-c levels. Supplementation of 2 g per day of phytosterols reduced TC and LDL-c by 8.2 and 9.3%^38^, but the effect of phytosterol supplementation in reducing LDL-c can be influenced by some factors, among them the time and frequency of administration, if given as a single or fractionated dose and the occasion of ingestion related to the type of meal. The best effect of supplementation is seen when administered with main meals (lunch and dinner) and divided into twice a day^38^. In the present study, phytosterols were added in the form of capsules along with the main meals, in 2 takes. In addition, the responsiveness to plant sterols and their bioavailability also depends on age and gender and are determined by nutrigenetic differences in metabolic factors that affect cholesterol uptake, CYP7A1 cytochrome activity, and APOE gene expression^38^.

Studies reveal that decreasing LDL-c and increasing HDL-c leads to a regression of atherosclerotic lesion^39^, although the latter has not proven event reduction. In recent decades, the cardioprotective role of HDL has been extensively investigated and directly associated with reverse cholesterol transport^40^. Such removal of cholesterol from the vessel wall followed by transport to the liver and excretion to the bile represents an essential biological function of HDL^40^.

Structural analysis and composition of the HDL particles can provide essential information for identifying HDL subfractions exhibiting specific biological functions, as well as for identifying functionally-deficient subfractions. Our findings show a trend to lower levels of intermediate HDL-7 subfraction with phytosterol supplementation, and both interventions reduced the atherogenic ILD-B subfraction compared with baseline. These subfractions of intermediate-density lipoproteins may contribute to residual cholesterol risk, as shown in the JUPITER study^41^, as well as seen in the phase 2 EQUATOR study, with evolocumab^42^. High serum concentrations of LDL-c are a risk factor for ASCVD, however, there are LDL particles in a variety of sizes that can differentially affect the progression of ASCVD^43^.

Smaller, denser LDL particles are considered more atherogenic and therefore a significant risk factor for ASCVD and cardiovascular events^44^. Atherogenic properties of small and dense LDL particles increase transport to artery wall ^45,46^, binding to arterial wall proteoglycans, the susceptibility to oxidation, and are absorbed by macrophages as part of the plaque-forming process^47^, with decreased binding to LDL receptor^45,46^.These findings are due to the greater affinity of the LDL receptor for larger and floating particles, while small and dense LDL particles have longer residence time in the circulation due to their lower affinity to the LDL receptor^48^.

As secondary outcome, both interventions showed a trend in the reduction in small and dense LDL particles. In studies such as MESA^49^, ARIC^50^, and *Quebec Cardiovascular Study*^51^ small, dense LDL particles had a higher risk of coronary heart disease. In another study, patients who experienced stroke presented smaller sizes of LDL and these small, dense LDL particles were predictors of stroke risk and stroke mortality^52^.

In our study, we correlated the classical lipid profile with the subfractions of LDL and HDL lipoproteins, using Lipoprint or Sampson equation, and we observed that the methods were complimentary. Reduced HDL-c levels correlate with small, dense particle size^53^. Increasing the HDL-c and HDL size may promote protection to ASCVD; additionally, there was correlation between triglycerides with the size of LDL (small and dense)^54^.

In the Z-scan and UV–vis studies, we observed that the θ value was strongly correlated with the absorption value at the 484 nm wavelength, which indicates that the greater the θ values, the absorption, and the number of carotenoids in the LDL particles are greater. This fact implies that LDL particles are more protected against oxidation as their θ values increase. None of the Z-scan and UV–vis analyses showed statistically significant differences in the quality of the LDL particles, showing no benefit, but also no harm to the functional properties of the particle. In other words, independent of other benefits of phytosterols ingestion, the quality of the LDL particles was the same in all the groups investigated. The differentiation of subfractions of non-atherogenic LDL (LDL-1 and LDL-2) and atherogenic LDL (LDL-3 to LDL-7) could not be performed using this technique.

A meta-analysis^55^ evaluating the effects of consumption of 1.5 to 2.0 g/day of phytosterols in hypercholesterolemia found a reduction on TC and LDL-c regardless of how phytosterols were consumed. Unlike our work, other studies that used the diet to assess the effect of phytosterols are based on administering food supplemented with phytosterols, the most common being margarines, yogurts, and cereals. Other studies with ingestion of capsule or tablet supplements with stanols or sterols of up to 1.8 mg/day showed a reduction in LDL-c, but Ottestad et al.^56^ using doses of 2 g/day did not encounter reduction in LDL-cholesterol levels. These findings emphasize the need to choose an appropriate dose and administration system to achieve optimal cholesterol-lowering effects.

In summary, this pilot-study, showed that phytosterol added to the diet demonstrated beneficial effects on HDL-7 subfraction, with no effect on the quality or oxidation of LDL particles. Secondary outcomes showed that both interventions reduced atherogenic IDL-B.

### Study strengths and limitations

To our knowledge this study is the first assessing, by different methods, the effects of phytosterol supplementation on functional properties of HDL and LDL particles, particle size, and the oxidative status of LDL in the primary prevention setting in participants with hypercholesterolemia.

This is a pilot-type study, with a small sample size, and the findings of this study need to be confirmed with larger samples, and chance findings cannot be ruled out.

Military police from ROTA are a special battalion of armed policeman that face danger every day, without time for meals, or leisure, and the data suggests that they did not show a healthy diet compliance. In addition, they reported a very low intake of phytosterols from the diet.

## Conclusions

In this pilot study, phytosterol added to the diet demonstrated small beneficial effects on HDL-7 subfraction, compared with diet alone. Both interventions reduced atherogenic IDL-B, with no effect on the quality of LDL particles.

### Supplementary Information

Supplementary Information.

## Data Availability

The analyzed database is available from the corresponding author upon request.
