# Supplementary material for: Effects of phytosterol supplementation on lipoprotein subfractions and LDL particle quality
Source: Sci Rep. 2024 May 15;14:11108. doi: 10.1038/s41598-024-61897-4 (PMC11096344; doi:10.1038/s41598-024-61897-4)
Supplement: Supplementary file 1 — Supplementary Information. [file 41598_2024_61897_MOESM1_ESM.docx]

**SUPPLEMENTARY INFORMATION**

**EFFECTS OF PHYTOSTEROL SUPPLEMENTATION ON LIPOPROTEIN SUBFRACTIONS AND LDL PARTICLE QUALITY**

Valeria Arruda Machado^1^, Angela Rocio Niño Santisteban^2^, Celma Muniz Martins^1^, Nagila Raquel Teixeira Damasceno^3^, Francisco A. Fonseca^1,2^, Antonio M. Figueiredo Neto^2^, Maria Cristina Izar^1,2^*.

^1^Cardiology Division, Department of Medicine, Federal University of Sao Paulo, Sao Paulo, SP, Brazil

^2^National Institute of Complex Fluids, Institute of Physics, University of São Paulo, São Paulo, SP, Brazil.

^3^Nutrition Department, Faculty of Public Health, University of São Paulo, São Paulo, SP, Brazil.

*Corresponding Author:

Maria Cristina Izar, MD, PhD.

Adjunct Professor of Medicine, Cardiology Division, Department of Medicine, Federal University of São Paulo, São Paulo, SP, Brazil

Rua José de Magalhães, 340 - São Paulo, SP, Brazil

Phone/fax: +55 11- 50879423

E-mail: cristina.izar@unifesp.br; mcoizar@cardiol.br

**Supplemental Table 1**. Biochemical profile of the participants, according to interventions

| Variables | Baseline (N=23) | Diet  (N=23) | Diet + Phytosterol  (N=23) | P-value |
| --- | --- | --- | --- | --- |
| Glycemia, mmol/L | 5.05±0.55 | 5.38±0.50 | 5.27±0.50 | 0.06 |
| HbA1c, % | 5.6 (0.3) | 5.7 (0.3) | 5.6 (0.4) | 0.94 |
| TC, mmol/L | 6.04 ± 0.62 | 5.98 ± 0.59 | 5.70 ± 0.62 | 0.04 |
| HDL-C, mmol/L | 1.04 (0.23) | 1.14 (0.23) | 1.11 (0.34) | 0.01 |
| Non-HDL-C, mmol/L | 4.92 ± 0.65 | 4.79 ± 0.59 | 4.56 ± 0.59 | 0.11 |
| LDL-C, mmol/L | 4.09 ± 0.54 | 4.04 ± 0.54 | 3.83 ± 0.70 | 0.07 |
| TG, mmol/L | 1.49 (0.97) | 1.61 (0.86) | 1.35 (0.72) | 0.29 |
| Urea, mg/dL | 33.0 ± 7.0 | 34.9 ± 6.0 | 33.0 ± 6.0 | 0.22 |
| Creatinine, mmol/L | 90.2 (20.3) | 87.5 (14.1) | 88.4 (15.9) | 0.46 |
| AST, U/L | 21 (5) | 21 (13) | 19 (6) | 0.53 |
| ALT, U/L | 27 ± 9 | 24 ± 7 | 25 ± 9 | 0.42 |
| GGT, U/L | 33 (25) | 31 (21) | 32 (25) | 0.86 |
| CK, U/L | 172 (191) | 196 (291) | 188 (249) | 0.83 |
| hs-CRP, mg/L | 0.12 (0.20) | 0.10 (0.10) | 0.10 (0.30) | 0.69 |
| TSH, µIU/mL | 2 ± 1 | 2 ± 1 | 3 ± 1 | 0.49 |
| FT4, ng/dL | 1 (0.15) | 1 (0.11) | 0.97 (0.15) | 0.16 |

Numerical variables presented as mean (SD) or median (AIQ) and compared by ANOVA and post Newman-Keuls test or Friedman and Dunn’s post test.

Baseline TC > Diet + phytosterol TC, p=0.04; baseline HDL-c < Diet + phytosterol HDL-c, p=0.02

AIQ, amplitude interquartile = Q_75%_ - Q_25%_; ALT, alanine aminotransferase; AST, aspartate aminotransferase; CK, creatine phosphokinase; HbA1c, glycated hemoglobin; HDL, high-density lipoprotein; hs-CRP, high-sensitivity C-reactive protein; IQ, interquartile range; LDL, low-density lipoprotein; SD, standard deviation; TC, total cholesterol; TG, triglycerides; TSH, thyroid stimulating hormone; FT4, free thyroxin.

**Legends for figures**

**Supplemental Figure 1.** Correlations between absorbance at wavelength 484 nm and θ, after twenty four weeks of treatment by crossover study.

**Supplemental Figure 2.** UV-visual spectroscopy wavelength absorbance spectrum of β-sitosterol in ethyl acetate as solvent.

Footnotes:

The spectrum at 180-400 nm corresponds to the β-sitosterol homologous molecule of cholesterol. Dilutions were carried out in concentrations from 0 to 2 g / mL.

**Supplemental Figure 1**


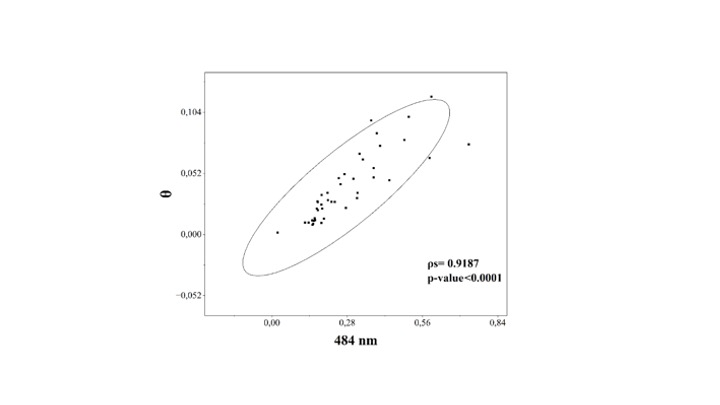


**Supplemental Figure 2**

**
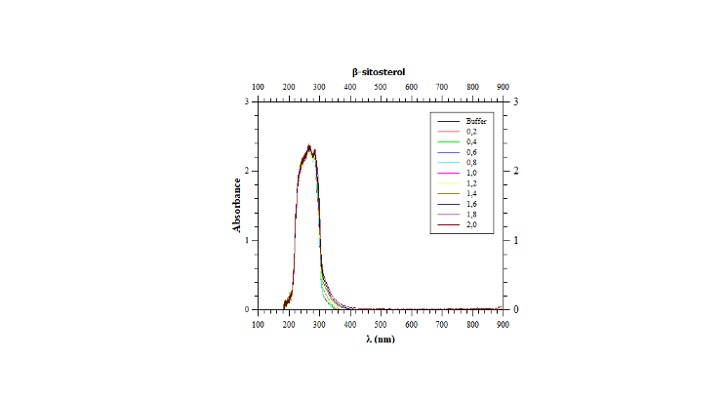
**
